# Experimental characterization of ${\text {H}}_2$/water multiphase flow in heterogeneous sandstone rock at the core scale relevant for underground hydrogen storage (UHS)

**DOI:** 10.1038/s41598-022-18759-8

**Published:** 2022-08-26

**Authors:** Maartje Boon, Hadi Hajibeygi

**Affiliations:** Faculty of Civil Engineering and Geosciences, Delft University of Technology, P.O. Box 5048, 2600 GA Delft, The Netherlands

**Keywords:** Hydrology, Energy storage

## Abstract

Geological porous reservoirs provide the volume capacity needed for large scale underground hydrogen storage (UHS). To effectively exploit these reservoirs for UHS, it is crucial to characterize the hydrogen transport properties inside porous rocks. In this work, for the first time in the community, we have performed ${\text {H}}_2$/water multiphase flow experiments at core scale under medical X-ray CT scanner. This has allowed us to directly image the complex transport properties of ${\text {H}}_2$ when it is injected or retracted from the porous rock. The important effective functions of capillary pressure and relative permeability are also measured, for both drainage and imbibition. The capillary pressure measurements are combined with MICP data to derive a receding contact angle for the ${\text {H}}_2$/water/sandstone rock system. The rock core sample is a heterogeneous Berea sandstone (17 cm long and 3.8 cm diameter). Our investigation reveals the interplay between gravitational, capillary, and viscous forces. More specifically, it illustrates complex displacement patterns in the rock, including gravity segregation, enhancement of spreading of ${\text {H}}_2$ due to capillary barriers, and the formation of fingers/channel during imbibition which lead to significant trapping of hydrogen. These findings shed new light on our fundamental understanding of the transport characteristics of ${\text {H}}_2$/water relevant for UHS.

## Introduction

To mitigate climate change a transition towards green energy systems, with renewable energy sources and large scale energy storage, is essential. Renewable energy sources such as solar and wind can generate clean electricity of which the surplus can be used to convert water into hydrogen through electrolysis^1^. Hydrogen is an attractive energy carrier because of its high specific energy capacity and its clean combustion products^2^. Large scale energy storage in the form of hydrogen will require volumes much beyond the capacities of surface-based facilities due to its low density^3^. Geological formations such as salt caverns, depleted hydrocarbon reservoirs and saline aquifers, are able to provide these gigantic volumes^4^. Hydrogen can be stored in salt caverns with a high degree of purity as it will have limited risk of contamination due to interactions with the existing reservoir fluid and rock salt^5^. Many geographical locations, however, will not have the required geology of evaporitic formations with suitable thickness and extent to create such caverns^1^. Porous reservoirs, on the other hand, are geographically abundant and can provide the volume capacity needed for large scale underground hydrogen storage (UHS)^1,6,7^.

Gasses such as ${\text {CH}}_4$, air, and ${\text {CO}}_2$ have been stored safely and successfully underground in depleted reservoirs and saline aquifers. During such gas storage operations, the injected gas displaces the existing reservoir fluids, and buoyantly rises until it reaches the impermeable caprock, after which it will spread laterally until a trap/anticline structure will prevent its lateral escape to allow reproduction^1^. The experience with UHS in such reservoirs is limited^8,9^. However, it is expected that the operation of UHS will be much different compared to UGS as the cyclic loading and frequency of injection and reproduction cycles will be determined by the intermittent green energy production. Furthermore, the purity of the gas stream reproduced from the reservoir will play a more important role as sensitivities towards hydrogen impurities in upstream facilities and applications are to be expected^10^. In addition to these operational differences, hydrogen’s low density and low viscosity could lead to complex interplay of gravitational, capillary and viscous forces, resulting in much different displacement patterns than observed for ${\text {CH}}_4$, air and ${\text {CO}}_2$, which could impact the reproduction.

In the case of underground hydrogen storage in saline aquifers, the injected hydrogen (or cushion gas) will initially displace the more viscous and denser reservoir brine during drainage, while during imbibition which involves the reproduction of the hydrogen, the brine will be displacing the less viscous and less dense hydrogen. Unfavourable viscosity contrasts, as observed during drainage, can lead to an unstable displacement front and the formation of complex fingering patterns^11^. Furthermore, density differences can lead to gravity override and the formation of a gravity tongue^12^. The displacement patterns are further complexified by rock structure heterogeneity which can lead to channeling of the flow predominantly along heterogeneity layers^11,13^. Both fingering and channeling can lead to uncontrolled lateral spreading resulting in pockets of unrecoverable hydrogen^1^.

To assess the feasibility of hydrogen storage in porous reservoirs, a good understanding of the movement of the hydrogen plume is needed during subsequent injection and reproduction cycles. The distribution of hydrogen in the reservoir will depend on the interaction of hydrogen with the existing brine and the reservoir rock. This interaction can be characterized with multiphase flow parameters such as relative permeability and capillary pressure^14–16^, which are important input parameters for reservoir simulators. These multiphase flow parameters depend on the wettability of the system, and the interplay between capillary, viscous and gravitational forces^15^. Capillary forces are responsible for the immobilization and trapping of the non-wetting phase, which is called residual trapping, resulting in hysteresis in the relative permeability and capillary pressure functions^17^.

Recently several methods have been used to characterize the wettability relevant to UHS including: (1) indirect contact angle measurements by combining capillary pressure measurements with mercury injection capillary pressure (MICP) data^18^, (2) the tilted plate experimental technique^19^, (3) the captive bubble cell approach^20–22^, (4) in-situ 3D micro-CT methods^22^, (5) microfluidics^23,24^. Water-wet conditions have been found in each of these studies with contact angles for the ${\text {H}}_2$/brine system ranging between [$0^{\circ }$ and $59.75^{\circ }$], with higher contact angles obtained for measurements carried out in real sandstone rock cores. Water wet conditions are favourable for UHS as the hydrogen will preferentially flow through the larger pores resulting in a higher relative permeability. This facilitates the injectivity of the reservoir, while the amount of capillary-trapped hydrogen will be smaller^25,26^. Furthermore, several of these studies showed that similar contact angles were derived for ${\text {H}}_2$, ${\text {CH}}_4$, ${\text {N}}_2$ and ${\text {CO}}_2$^21,23^. Despite similar wettability characteristics, very different multiphase flow parameters, such as relative permeability and capillary pressure, are to be expected for the ${\text {H}}_2$/water system compared to other gas/water systems due to the complex interplay of viscous, capillary and gravitational forces.

One approach to derive meaningful relative permeability and capillary pressure functions for UHS is by conducting pore network modeling^6^ based on the pore-scale interfacial characteristics such as contact angles^23^. Another approach is to measure these parameters directly during core-flood tests in the laboratory^14,15,17^. Relative permeability can be measured directly using unsteady^27,28^ and steady-state^15,17,29–31^ core-flood test techniques. Capillary pressure curves for the ${\text {H}}_2$/water/rock system can be derived from MICP data of small rock samples and using the Young-Laplace equation to convert the data from the Hg/air to the ${\text {H}}_2$/water system^14,18^. This requires the interfacial tension and contact angle of both systems. Capillary pressure can be measured directly for the fluid pair of interest and for larger rock cores using the semi-permeable disk technique^32–34^, the modified semi-dynamic technique^18,35,36^ and the steady-state technique presented by Pini et al.^14^, which uses the same experimental apparatus as used for the steady-state relative permeability measurement technique^15^. By combining the capillary pressure measurements for the ${\text {H}}_2$/water/rock system with MICP data of the Hg/air/rock system, receding contact angles for the ${\text {H}}_2$/water/rock system can be derived^18^.

Despite the importance of relative permeability and capillary pressure, to date, only the study of Yekta et al.^18^ has presented direct measurements of these parameters during drainage for the ${\text {H}}_2$/water/rock system. A Vosges sandstone was used for these experiments and the steady-state technique^29^ and modified semi-dynamic technique were applied^35,36^ to measure relative permeability and capillary pressure, respectively. The experiments were carried out to represent shallow (50 bar—20 $^{\circ }{\text {C}}$) and deep (100 bar—45 $^{\circ }{\text {C}}$) aquifers. The 3D saturation map, indicative of the complex flow behaviour of such systems, was not visualized. Instead, the mass balance method was used for the water saturation measurements. A few observations of the distribution of hydrogen during core-flood tests exists. Higgs et al.^22^ and Jha et al.^37^ used X-ray micro CT to visualize the distribution of hydrogen in initially brine saturated Gosford sandstone and Bentheimer sandstone, respectively. The latter study found an initial hydrogen saturation of up to 65$\%$ and a residual hydrogen saturation after imbibition of 41$\%$. The work of Al Yaseri et al.^38^ carried out core-flooding experiments on a larger Fontaineblue sandstone rock sample (5.45 cm long and 3.86 cm in diameter) for both hydrogen and nitrogen. The gas saturations were visualized during drainage and imbibition using Nuclear Magnetic Resonance (NMR) under pressure and temperature that represent shallow reservoirs. Results show significantly lower initial and residual hydrogen saturations, 4$\%$ and 2$\%$ respectively, in comparison with nitrogen where the initial and residual saturations were 15$\%$ and 8$\%$, respectively, for the same capillary number.

In this study, the flow behaviour of hydrogen in initially water saturated rock is visualized during a core-flood test carried out for a heterogeneous Berea Sandstone core (17 cm in length and 3.8 cm in diameter), for both drainage and imbibition, at $18^{\circ }{\text {C}}$ and 100 bar, using a medical X-ray CT scanner. Furthermore, relative permeability and capillary pressure curves are derived. The initial and residual saturations are measured and presented in an IR plot. The wettabiltity of the system is characterized by combining the capillary pressure measurements with MICP data. This study is the first of its kind, and sheds new light on our understanding of the mm-cm scale transport characteristics of hydrogen in porous rocks, relevant to underground hydrogen storage.

## Material and methods

In this study, relative permeability, capillary pressure and residual trapping are measured for the ${\text {H}}_2$/water system by carrying out core-flood tests on a heterogeneous Berea Sandstone core for both drainage and imbibition. A medical X-ray CT scanner is used to visualize the distribution of hydrogen in the rock core. The experimental apparatus and procedure are based on the work of Krevor et al.^15^ and Pini et al.^14^. Receding contact angles are derived by combining the capillary pressure measurements with MICP data^18^.

### Rocks and fluids

**Figure 1:**
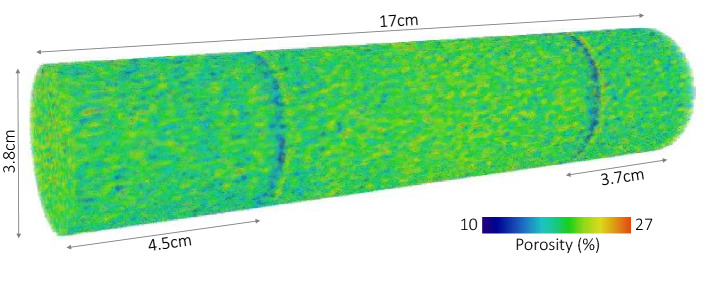
3D porosity map of the core. The two low porosity bands are clearly visible.

Degassed tap water, hydrogen gas, and a Berea (Liver) sandstone rock core are used in the experiments. The hydrogen gas consists of 99.99 mol% purity ${\text {H}}_2$ produced by Linde-gas Company. The Berea sandstone rock core is 17 cm in length and 3.8 cm in diameter. The sample is untreated and consist mainly out of quartz (95%)^20^. Two low porosity bands cut across the core at 4.5 cm and 13.3 cm from the inlet of the core. The permeability (*k*) of the sample is 203 mD ($2\times 10^{-13}\;{\text {m}}^2$) and the porosity is 19.7%. Figure 1 shows the 3D porosity map of the core. The two low porosity bands are clearly visible.

The experiments are carried out at $18^{\circ }\;{\text {C}}$ and a pressure of 100 bar. At these conditions, the viscosity of hydrogen ($\mu _{H_2}$) and water ($\mu _{water}$) are $8.88 \times 10^{-6}$ Pa s and $1.049 \times 10^{-3}\;{\text {Pa s}}$, while the densities ($\rho _{H_2}$ and $\rho _{water}$) are $7.85\;{\text {kg/m}}^3$ and $1003.1\;{\text {kg/m}}^3$, respectively^39^. This results in a density difference ($\Delta \rho$) of $995.25\;{\text {kg/m}}^3$. The interfacial tension ($\gamma$) is 71.9 mN/m^40^. The injection rate for the relative permeability measurements is 5 ml/min, which corresponds to a superficial velocity ($\nu$) of $7.35 \times 10^{-5}\;{\text {m/s}}$.

A complex interplay of viscous, capillary and gravitational forces is expected for the ${\text {H}}_2$/water/rock system. To describe the different force balances, the pore scale capillary number (*Ca*), the macroscopic capillary number ($N_{cv}$), and the gravity number ($N_{gv}$) are used, and defined as^41^

1 $$\begin{aligned} Ca= & {} \frac{\nu \mu _{H_2}}{\gamma }, \end{aligned}$$

2 $$\begin{aligned} N_{cv}= & {} \frac{kLp_{c}^*}{H^2\mu _{H_2}\nu }, \end{aligned}$$

and

3 $$\begin{aligned} N_{gv}=\frac{\Delta \rho g k L}{H \mu _{H_2}\nu }. \end{aligned}$$

where, L is the length of the core [m], H is the height of the core [m], $p_{c}^*$ is a characteristic capillary pressure for which the entry pressure ($p_e=8995$Pa) is used, and *g* is the acceleration of gravity [m/${\text {s}}^2$]. For the conditions of the experiment a *Ca* of $9.1\times 10^{-9}$ and $N_{cv}$ of $3.24\times 10^{2}$ is obtained, pointing towards a capillary dominated regime^41^. The $N_{gv}$ is calculated as $1.3\times 10^{1}$ showing that gravitational forces dominate over viscous forces^41^. The bond number, i.e.,

4 $$\begin{aligned} N_{B}=\frac{N_{gv}}{N_{cv}}=\frac{\Delta \rho g H}{p_{c}^*}, \end{aligned}$$

determines how the gravitational forces relate to the capillary forces. The $N_B$ is $4.1\times 10^{-2}$ indicating that the capillary forces dominate over the gravitational forces.

At the mm-scale the two-phase displacement behaviour highly depends on the interplay of viscous and capillary forces which can lead to three different displacement regimes: viscous fingering, capillary fingering and stable displacement. This can be characterized by the pore-scale capillary number (Eq. 1) using the viscosity of the displacing phase, and the viscosity ratio^42^, $M=\frac{\mu _{2}}{\mu _{1}}$, where $\mu _{1}$ is the viscosity of the displacing phase and $\mu _{2}$ the viscosity of the displaced phase^43^. Using these definitions the *Ca* and *M* of the drainage and imbibition, corresponding to the flowrate of 5ml/min are, respectively, $Ca_{drain} =9.1\times 10^{-9}$ and $M_{drain}=1.2\times 10^{2}$ and $Ca_{imb} =1.1\times 10^{-6}$ and $M_{imb}=8.5\times 10^{-3}$. Therefore, based on the phase diagrams for drainage and imbibition presented in the work of Guo and Aryana^43^, the capillary fingering displacement regime can be expected for both drainage and imbibition.

### Experimental apparatus

**Figure 2:**
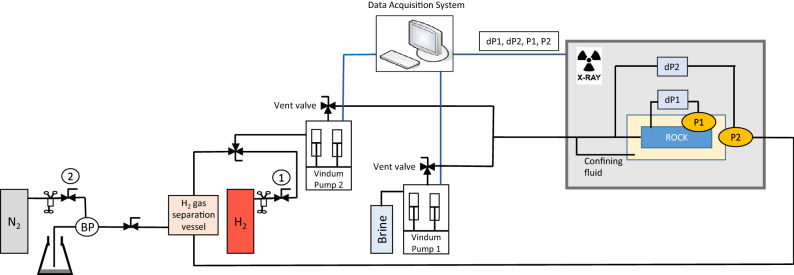
Schematic of experimental apparatus.

A schematic of the experimental apparatus can be seen in Fig. 2. The outer surface of the rock sample is covered with a 5mm thick layer of epoxy resin to form a barrier for the fluids. The rock sample is placed inside a peek coreholder. The confining pressure in the coreholder is created with a special connection to the inlet line. The pressure is measured at four locations along the core: the inlet, the outlet, 2.25 cm from the inlet, and 2.25 cm from the outlet. The absolute pressure is measured at the outlet and at 2.25 cm from the outlet with two pressure transducers (GE 5000 series—0.2 premium up to 150 bar). Two pressure differential (dP) devices (Deltabar S PMD75 Endress + Hauser) are used to obtain the pressures at the other two locations. To be able to continuously co-inject water and hydrogen into the rock core two pulse-free high-precision piston pumps (Vindum) are used, one for hydrogen and one for water. The inlet end-cap consists of one inlet port located in the center of the end-cap through which both hydrogen and water are injected. The inlet line between the pumps and the inlet end-cap is kept long to equilibrate the water and hydrogen before entering the core. The effluent is collected in a 150 ml collection vessel. The top of the collection vessel is connected to the hydrogen pump to recirculate the hydrogen in the experiment. The bottom of the collection vessel is connected to a back-pressure regulator. The back-pressure is regulated with a large nitrogen cylinder to maintain the pressure of 100 bar in the experiments. The effluent water is collected in a waste container. Fluid saturations are measured using a medical X-ray CT scanner. The slice thickness of the scans is 0.6 mm, while the resolution in the other two dimensions is 0.19 mm resulting in a voxel size of $0.19\times 0.19\times 0.6$ ${\text {mm}}^2$. A voltage of 140 kV and a current of 250 mA is used. Each of the scans is taken in three-fold and averaged to reduce the random error in CT number. In this experimental configuration, the hydrogen phase is recirculated, while the water pumps are refilled from a large container with degassed tap water. The water is equilibrated with the hydrogen gas in the long inlet lines. As a consequence, the volume of hydrogen gas in the system slowly decreases with time. To ensure that enough hydrogen gas stays within the system, the volume of hydrogen in the system is refilled to a volume of 90ml at the beginning and end of each day.

### Experimental procedure

The experimental techniques used to measure steady-state relative permeability and capillary pressure are based on the techniques described in Krevor et al.^15^ and Pini et al.^14^, respectively. The experiment consists of four stages: (1) background scans and absolute permeability test, (2) drainage relative permeability measurements, (3) drainage capillary pressure measurements, (4) imbibition relative permeability and residual trapping measurements. Each of these stages are described in detail below.

#### Background scans and absolute permeability test

At the start of the experiment background scans are taken of the dry core at ambient pressure. Next the core is saturated with hydrogen at a pressure of 100 bar. This is done by injecting hydrogen while simultaneously slowly increasing the backpressure to the experimental pressure of 100 bar. Once the experimental pressure of 100 bar is reached background scans of the hydrogen saturated core are taken, after which the pressure is reduced back to ambient pressure. To completely saturate the core with water and remove all the gas, the system is first flushed with ${\text {CO}}_2$. Next, the outlet of the core is connected to a vacuum pump while injecting water at the inlet. Once the water reaches the vacuum pump, the outlet lines are connected to the back pressure regulator. The pressure is slowly increased to the experimental pressure of 100 bar and background scans of the water saturated core are taken. The background scans of the dry core and water saturated core are used to calculate a 3D porosity map according to

5 $$\begin{aligned} \phi = \frac{CT_{water}-CT_{dry}}{I_{water}-I_{air}}. \end{aligned}$$

Here, $CT_{water}$ and $CT_{dry}$ are the voxel level CT numbers [HU] for the water and dry core, respectively. $I_{water}$ and $I_{air}$ are the CT values [HU] that would have been obtained if water and air were scanned^15^. The permeability of different sections of the core, as well as the core as a whole, is determined by injecting water for a range of flow rates. Using these flow rates together with the obtained pressure drops in Darcy’s law allows for the calculation of permeability.

#### Drainage relative permeability measurements

The steady-state relative permeability of the hydrogen and water phase can be calculated based on the one-dimensional form of the extended Darcy’s law and the assumption of constant capillary pressure throughout the core^31^, i.e.,

6 $$\begin{aligned} q_i =-\frac{Akk_{r,i}}{\mu _i}\frac{\Delta P}{L}. \end{aligned}$$

Here, the subscript *i* indicates either the water or the hydrogen phase, $q_i$ is the phase injection rate [${\text {m}}^3$/s], *A* is the surface area of the inlet of the core [${\text {m}}^2$], *k* is the permeability [${\text {m}}^2$], $k_{r,i}$ is the phase relative permeability [–], $\mu _i$ is the phase viscosity [Pa s], and $\Delta P$ the pressure drop [Pa] across length *L* [m]. Relative permeability is a function of saturation. To obtain the relative permeability for a range of saturation values, the fractional flow of hydrogen is increased in a step-wise matter to a fractional flow of 100% hydrogen. Scans are taken for each fractional flow once steady-state is reached. For each fractional flow the 3D steady-state saturation map is calculated according to

7 $$\begin{aligned} S_{H_2} = \frac{CT_{exp}-CT_{water}}{CT_{H_2}-CT_{water}}, \end{aligned}$$

where, $CT_{exp}$ ,$CT_{H_2}$ are the voxel level CT numbers [HU] for the core at a particular step in the experiment, and the hydrogen saturated core, respectively. The experimental conditions of the drainage relative permeability measurements can be found in Table 1.

**Table 1:** Experimental conditions during each stage of the experiment.

	$q_T$ [ml/min]	$f_{H_2}$ pump	$f_w$ pump	$f_{H_2}$ core	$f_w$ core	$\Delta P$ [Pa]	$k_{rw}$	$k_{rH_2}$	$S_w (ave)$	$P_c$ [Pa]	$S_w$($p_c$)
**Drain **$k_r$											
1	5	0.2	0.8	0.19	0.81	61560	0.6790	0.0014	0.96		
2	5	0.3	0.7	0.29	0.71	60210	0.6074	0.0022	0.93		
3	5	0.4	0.6	0.39	0.61	62850	0.4988	0.0028	0.92		
4	5	0.5	0.5	0.50	0.50	64750	0.4035	0.0034	0.90		
5	5	0.7	0.3	0.70	0.30	60110	0.2608	0.0052	0.86		
6	5	0.8	0.2	0.80	0.20	47820	0.2185	0.0074	0.84	10840	0.82
7	5	0.9	0.1	0.90	0.10	42270	0.1236	0.0094	0.80	12260	0.79
8	5	0.95	0.05	0.95	0.050	28020	0.0932	0.015	0.77	11225	0.74
9	5	0.97	0.03	0.97	0.030	19870	0.0789	0.0216	0.71	13880	0.65
10	5	0.99	0.01	0.99	0.010	28480	0.0183	0.0154	0.69	12630	0.66
11	5	1	0	1	0	22750	0	0.0194	0.64	13460	0.66
**Drain **$p_c$											
1	5	1	0	1	0	22750		0.019	0.64	13460	0.66
2	7	1	0	1	0	20510		0.030	0.63	13040	0.65
3	10	1	0	1	0	22800		0.039	0.60	14440	0.64
4	15	1	0	1	0	25190		0.053	0.58	14970	0.60
5	20	1	0	1	0	28330		0.062	0.56	14660	0.57
6	30	1	0	1	0	28750		0.092	0.53	15460	0.54
**Imb 1**											
${\text {S}}_w$ (initial)	30	1	0	1	0	28750		0.092	0.53		
1	5	0.97	0.03	0.97	0.030	31510	0.050	0.014	0.64		
2	5	0.95	0.05	0.95	0.050	48650	0.054	0.0086	0.64		
3	5	0.9	0.1	0.90	0.10	88130	0.059	0.0045	0.66		
4	5	0.8	0.2	0.80	0.20	156600	0.067	0.0022	0.67		
5	5	0.7	0.3	0.70	0.30	228800	0.069	0.0014	0.68		
6	5	0.5	0.5	0.50	0.50	367270	0.071	0.00060	0.68		
7	5	0.3	0.7	0.29	0.71	487700	0.075	0.00027	0.68		
8	5	0.2	0.8	0.19	0.81	129620	0.32	0.00068	0.76		
**Imb 2**											
${\text {S}}_w$ (initial)	5	1	0	1	0	17000		0.026	0.62		
1	5	0.97	0.03	0.97	0.030	31070	0.050	0.014	0.70		
2	5	0.95	0.05	0.95	0.050	43160	0.061	0.010	0.70		
3	5	0.9	0.1	0.90	0.10	76990	0.068	0.0052	0.71		
4	5	0.8	0.2	0.80	0.20	133980	0.078	0.0026	0.72		
5	5	0.7	0.3	0.70	0.30	198390	0.079	0.0016	0.72		
6	5	0.5	0.5	0.50	0.50	322120	0.081	0.00069	0.71		
7	5	0.3	0.7	0.29	0.71	235400	0.16	0.00056	0.79		
8	5	0.2	0.8	0.19	0.81	268720	0.16	0.00033	0.78		
9	5	0.1	0.9	0.08	0.92	242860	0.19	0.00018	0.80		
10	5	0.05	0.95	0.03	0.97	212910	0.23	0.00010	0.81		
11	5	0.02	0.98	0.00	1.0	225210	0.23	0.000049	0.83		

where, $q_T$ is the total injection rate (ml/min), $f_{H_2}$ and $f_{w}$ are the fractional flow of hydrogen and water, respectively. The fractional flow injected from the pump is listed as well as the fractional flow that is obtained in the core once the water has equilibrated with hydrogen. The amount of dissolved ${\text {H}}_2$ for the conditions of the experiment is calculated using Henry’s law with $K_{H}^{Pc}=1300 \frac{L\;{\text {atm}}}{\text {mol}}$^44^. $\Delta$P is measured between the pressure tap at 2.25 cm from inlet and the pressure tap at 17 cm from the inlet (outlet). Please note that drainage ${\text {p}}_c$(1) is the same measurement as drain $\text {k}_r$ (11).

#### Drainage capillary pressure measurements

The technique presented in Pini et al.^14^ measures water pressure at the outlet and gas pressure at the inlet. In our experimental configuration, water pressure is measured at the outlet, while the pressure in the hydrogen phase is measured at the inlet, as well as the two pressure taps at 2.25 cm from the inlet and outlet^36^. At steady-state, during the injection of 100% hydrogen, no more water is produced from the core, and therefore the assumption can be made that no gradient exists in the water pressure. Therefore, the water pressure will be the same everywhere in the core, and the measured water pressure at the outlet can be used to obtain the capillary pressure ($p_c$) at the three locations in the core where the hydrogen pressure is measured. The capillary pressure is defined as,

8 $$\begin{aligned} p_c=p_{nw}-p_w, \end{aligned}$$

where, $p_{nw}$ and $p_{w}$ are the pressure in the non-wetting and wetting phase, respectively, which in our case are hydrogen and water. To calculate the capillary pressure for a range of saturation values the flow-rate of the 100% hydrogen injection is step-wise increased. For each flow-rate, scans are taken once steady-state is reached. The slice average saturation at the location of the capillary pressure measurement can be calculated using Eq. (7). The experimental conditions of the drainage capillary pressure measurements can be found in Table 1. To extend the capillary pressure curve over the full range of water saturations, and to derive the receding contact angle of the ${\text {H}}_2$/water system, the capillary pressure measurements were combined with MICP data of another Berea (Liver) sandstone rock core that was used in the study of Ni et al.^17^ which had similar porosity (19%) and permeability (200.4 mD) as the Berea (Liver) used in this study. The Hg/air capillary pressure measurements can be converted to the ${\text {H}}_2$/water system using the Young-Laplace scaling^18^, i.e.,

9 $$\begin{aligned} p_{c,H_2/water}=p_{c,Hg/air}\frac{\gamma _{H_2/water} \cos (\theta _{H_2/water})}{\gamma _{Hg/air}\cos (\theta _{Hg/air})} \end{aligned}$$

Here, $\gamma$ is the interfacial tension [mN/m], $\theta$ is the contact angle [$^\circ$]. By fitting Eq. (8) to the capillary pressure measurements, the receding contact angle of the ${\text {H}}_2$/water/Berea system can be determined. The interfacial tension and contact angle of the Hg/air system are taken to be 485 mN/m and $140^\circ$, respectively. The interfacial tension of the ${\text {H}}_2$/water system is taken to be 71.9 mN/m^40^.

#### Imbibition relative permeability measurements

The imbibition steady-state relative permeability measurements were carried out twice for two different initial hydrogen saturations. For the first imbibition measurements (Imbibition 1), the saturation in the core obtained at the last point of the drainage capillary pressure measurements was the starting point for imbibition. For the second imbibition measurements (Imbibition 2), 100% hydrogen was injected into the water saturated core at an injection rate of 5ml/min until steady-state was reached, before starting imbibition. This resulted in a lower initial hydrogen saturation ($S_{gi}$) as the starting point for imbibition compared to the first imbibition measurements. The steady-state imbibition relative permeability was calculated using Eq. (6). To calculate the imbibition relative permeability for a range of saturation values the fractional flow of water was increased in a step-wise matter to a fractional flow of 100% water. At steady-state, for each fractional flow, scans were taken and saturations were calculated according to Eq. (7). The experimental conditions during each step of the imbibition experiments can be found in Table 1.

The residual gas saturation ($S_{gr}$) is determined from the steady-state saturation obtained during the 100% water fractional flow. The trapping ability of the rock is characterized with the linear trapping coefficient (*A*)^17^, defined as

10 $$\begin{aligned} A=\frac{S_{gr}}{S_{gi}}. \end{aligned}$$

High trapping coefficients correspond to a high trapping ability. Low trapping coefficients are favourable for UHS.

#### Safety

Safety aspects are very important when working with hydrogen. The following safety measures are in place: 1. The room is very well ventilated. 2. A hydrogen monitor is installed next to the experiment. 3. The hydrogen cylinder is closed at all times, except for when refilling the hydrogen pump. 4. A maximum volume of 90ml of hydrogen is used which is recirculated in a closed flow loop. This volume is large enough to carry out the experiments, but small enough to pose limited safety risks in a well ventilated lab. 5. At the end of the experiment the hydrogen is discarded into the fume-hood.

## Results

### Flow behaviour of the H_2_/water system

**Figure 3:**
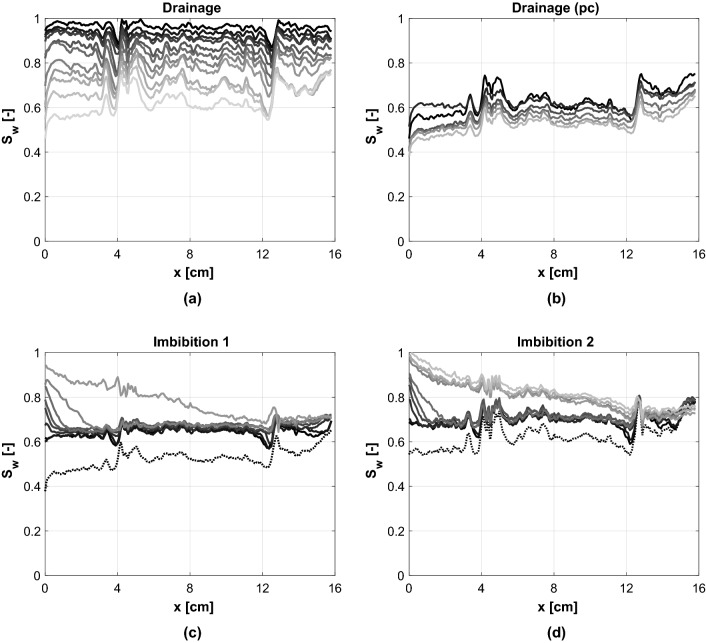
Steady-state saturation profiles along the length of the core. Each subplot indicates a different stage of the experiment. (**a**) Drainage relative permeability measurements. The water saturation is decreasing with increasing fractional flow of ${\text {H}}_2$. (**b**) Drainage capillary pressure measurements. 100% ${\text {H}}_2$ is injected at different flow-rates. The water saturation is decreasing with increasing flow-rate. (**c**, **d**) Imbibition relative permeability measurements for Imbibition 1 and Imbibition 2, respectively. The water saturation increases with decreasing fractional flow of ${\text {H}}_2$. The dotted line in the saturation profiles of Imbibition 1 and 2 shows the starting point of imbibition. Table 1 shows the experimental conditions during each stage of the experiment.

The steady-state saturation profiles along the length of the core, for each step during the drainage relative permeability stage of the experiment, are presented in Fig. 3a. The saturation stays relatively constant with distance, however, higher ${\text {H}}_2$ saturations can be observed before the low porosity bands which act as capillary barriers.

**Figure 4:**
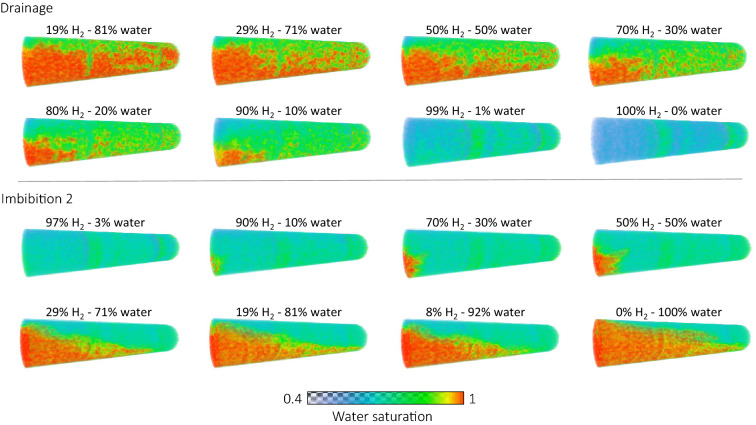
Steady-state water saturation maps for each step during Drainage and Imbibition 2.

From the 3D drainage saturation maps which can be seen in Fig. 4 it can be seen that at the inlet the hydrogen flows mainly at the top of the core due to gravity segregation. The low porosity zone at 4.5 cm from the inlet acts as a capillary barrier forcing the hydrogen to spread towards the bottom of the core. As a result the hydrogen is spread more evenly throughout the rest of the core and meaningful multiphase flow parameters can be derived for this region.

The drainage relative permeability stage was followed by drainage capillary pressure measurements of which the saturation profiles can be seen in Fig. 3b. The profile with the lowest water saturation was obtained during the last capillary pressure measurement, and formed the starting point of imbibition.

**Figure 5:**
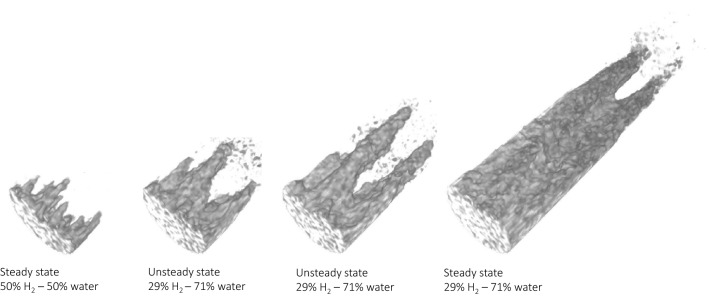
Steady-state high water saturation fingers evolving into a high water saturation channel during Imbibition 2.

The imbibition relative permeability stage of the experiment was carried out twice. Imbibiton 1 took place immediately after the last capillary pressure measurement. While Imibition 2 started after injection of 100% hydrogen at 5 ml/min into a water saturated core until steady-state was reached. Figure 3c,d show the steady-state saturation profiles for each step for Imbibition 1 and Imbibition 2, respectively. The dotted line shows the saturation at the start of imbibition. It can be seen that the initial ${\text {H}}_2$ saturation was higher for Imbibition 1 compared to Imbibition 2. For both Imbibition 1 and 2, during the first steps of the imbibiton process the steady-state saturation profiles are constant along the core, however, the water saturation is higher near the inlet. With each step, at steady-state, the water saturation at the inlet is increasing and the region of increased water saturation reaches further down the core. Initially this evolves gradually, however, for both Imbibition 1 and 2, a big change can be observed where the increase in saturation is higher and the region of increased saturation reaches much further down the core. A closer look at the 3D saturation maps, Fig. 4 Imbibition 2, shows that the initial increase in the steady-state saturation profile at the inlet was caused by the formation of fingers which gradually evolved with each step of the experiment. The big change in the steady-state saturation profile is observed when the fingers evolve into a channel as can be seen in Fig. 5. This happened at a fractional flow of 19% hydrogen and 81% water for Imbibition 1 and for a fractional flow of 29% hydrogen and 71% water for Imbibition 2.

Despite the low solubility of ${\text {H}}_2$ in water and the long inlet lines that were used to equilibrate the water with the ${\text {H}}_2$, it is likely that the observed behaviour is caused by the fact that the injected water was not fully equilibrated with the hydrogen. As a result miscible displacement takes place, where hydrogen is dissolved into the brine which, in combination with capillary fingering, results in the formation of high water saturation fingers. For the low water fractional flows the fingers grow at a very low rate and do not impact the overall flow behaviour, therefore a “pseudo” steady-state is reached and the relative permeability measurements can be made.

However, for high water fractional flows, the water saturation and, consequently, the relative permeability of the water phase in the fingers increases, and a preferential flow path for the water phase is created. In this case, the flow becomes more and more channelized and the fingers evolve into a channel. The pressure drop across the core stabilizes once the channel cuts through the core. However, water is now preferentially flowing through the channel and no meaningful relative permeability measurements can be made.

In the case of miscible displacement in homogeneous porous media, when the injected fluid is more viscous than the ambient fluid, a single dispersive front is to be expected^13^. The work of Loggia et al.^45^ showed that in layered heterogeneous media, a single dispersive front is attained when the viscosity ratio is larger than the ratio of permeabilities, while channelling is observed when the viscosity ratio is smaller than the ratio of permeabilities. In our case, at the inlet, imbibition starts with a homogeneous (relative) permeability field as the hydrogen is evenly distributed. During each step of the imbibition stage, the (relative) permeability field for the water phase becomes more heterogeneous near the inlet due to the high water saturation fingers formed by dissolution of hydrogen. Furthermore, the viscosity difference between the injected fluid and the ambient fluid becomes smaller because more hydrogen has been removed from the core. This results into a similar situation as described in the work of Loggia et al.^45^. Once the relative permeability ratio for the water phase is larger than the viscosity ratio, channels will start to form. The inital water saturation was lower for Imbibition 1, and therefore a higher water fractional flow was needed to reach the situation that allowed for channel formation, compared to Imbibition 2. This shows that, next to fingering due to unstable displacement during the drainage phase^1^, also channeling during the imbibition phase can result in pockets of unrecoverable hydrogen. In future work, we aim to model the presented experimental study to provide the theoretical understanding behind these observations.

### Relative permeability and capillary pressure measurements

Pressure measurements are taken at four locations along the core. Therefore, relative permeability can be calculated for different sections, and capillary pressure can be calculated for three locations of the core. However, no meaningful multi-phase flow parameters can be obtained for the region at the inlet where the flow of hydrogen and water are segregated. Therefore, the relative permeability measurements presented in this paper are made for the section between 2.25 cm from the inlet and 17 cm from the inlet (outlet), while the capillary pressure measurements are made for the location at 2.25 cm from the outlet.

**Figure 6:**
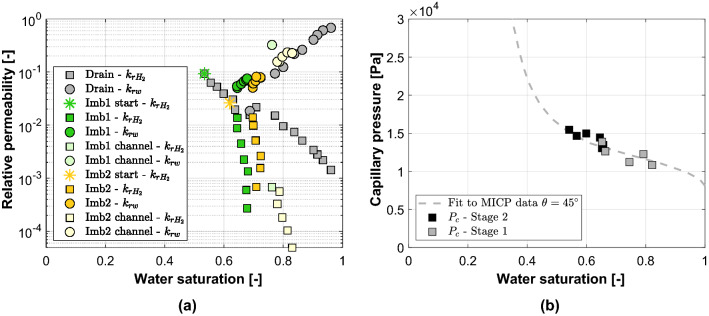
(**a**) Relative permeability curves—Drainage and imbibition. The circles indicate the water relative permeability measurements while the squares are the ${\text {H}}_2$ relative permeability measurements. In grey are the results for drainage, while in green and yellow the results for Imbibition 1 and 2 are presented, respectively. The lighter shaded green and yellow indicate the measurements that were taken after channel formation occurred. (**b**) Capillary pressure curve—Drainage. The dashed line shows the fit to MICP data obtained for a Berea (Liver) sandstone rock core with similar permeability and porosity presented in Ni et al.^17^. A receding contact angle of $45^{\circ }$ is derived. The black squares are the capillary pressures measured during stage 2: Drainage capillary pressure measurements. The grey squares are the capillary pressures measured during Stage 1: Drainage relative permeability measurements.

The relative permeability curves can be seen in Fig. 6a. It can be seen that the cross-over point between the relative permeability curves of both phases is low, indicating that extensive interference between the two phases exists, making it more difficult for both phases to flow. Furthermore, significant hysteresis between the drainage and imbibition curves can be observed for the hydrogen phase, while hysteresis is much less apparent for the water phase. The difference in the relative permeability curves for Imbibition 1 and 2 is the result of the different initial saturation at the start of imbibition. As such, the two imbibition curves form two different scanning curves. The imbibition relative permeability measurements taken after channel formation occurred do not provide meaningful relative permeability data and are indicated by the light shaded markers in Fig. 6a.

The drainage capillary pressure measurements can be seen in Fig. 6b. Capillary pressure measurements were made during Stage 2 where 100% ${\text {H}}_2$ was injected using a similar approach as described in Pini et al.^14^. In addition, capillary pressure measurements were also made during the drainage relative permeability stage. In our experimental configuration, ${\text {H}}_2$ pressure is measured at the pressure tap at 2.25 cm from the outlet during the drainage relative permeability stage (Stage 1). The water pressure at this location was calculated from the water pressure at the outlet, using the average pressure gradient in the core. The capillary pressure measurements made during Stage 1 are indicated in grey. By combining our capillary pressure measurements with MICP data for the Hg/air system obtained for a Berea (Liver) sandstone rock core with similar permeability and porosity presented in Ni et al.^17^, a receding contact angle of $45^{\circ }$ was derived. This is in agreement with the contact angles found for the ${\text {H}}_2$/brine/rock system in literature^22^. A comparison between the drainage relative permeability and capillary pressure curves of this study and the study of Yekta et al.^18^ can be found in the “Supplementary information”.

### Residual trapping

**Figure 7:**
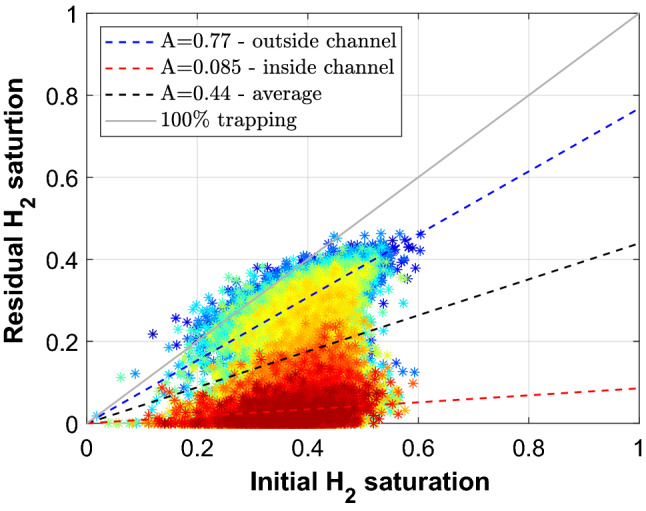
IR plot. Initial ${\text {H}}_2$ saturation at the start of Imbibition 2 versus the trapped ${\text {H}}_2$ saturation at the end of Imbibition 2. The colored stars are the voxel level ${\text {H}}_2$ saturation. The color correspond to vertical location in the core. Blue is at the top of the core and red is at the bottom of the core where the channel formed. The blue, black and red dashed lines are the best-fit linear trapping relationships for the top of the core (outside channel), the entire core, and the bottom of the core (inside channel), respectively. The grey line indicates the 100% trapping line.

Figure 7 shows the voxel level residual ${\text {H}}_2$ saturation as a function of the initial ${\text {H}}_2$ saturation which is the saturation at the start of Imbibition 2. The different colors show the vertical location in the core: blue is at the top of the core, red is at the bottom of the core where the channel formed. The blue, black and red dashed lines are the best-fit linear trapping relationships for the top of the core (outside channel), the entire core, and the bottom of the core (inside channel), respectively. The grey line indicates the 100% trapping line. The water preferentially flows through the channel and is able to sweep most of the hydrogen in the channel (A = 0.085). However, outside the channel the amount of trapping is very high (A = 0.77). The average linear trapping coefficient of the core is 0.44, suggesting that due to fingering and channel flow a significant amount of hydrogen loss can occur.

## Conclusion

To characterize the flow behaviour for the ${\text {H}}_2$/water system a core flood test was carried out, at $18\ {^{\circ }{\text {C}}}$ and 100 bar, on a Berea sandstone rock core, 17 cm in length and 3.8 cm in diameter, for both drainage and imbibition, during which relative permeability and capillary pressure were measured. The obtained multiphase flow parameters provide important input parameters for reservoir simulators to model the behaviour of the hydrogen plume in the reservoir during UHS. The saturation distribution in each step of the experiment was visualized with the use of a medical X-ray CT scanner. By combining the capillary pressure measurements with MICP data a receding contact angle of $45^{\circ }$ was derived. This is in agreement with in-situ contact angle measurements using micro CT on sandstone rock cores^22^.

Our work shows that the interplay between gravitational, capillary and viscous forces can lead to complex displacement patterns during UHS in reservoir rock. The high density contrast between the ${\text {H}}_2$ and water phase can result in gravity segregation. Capillary barriers can counteract this effect, and enhance the spreading of hydrogen. High water saturation fingers can form during imbibition resulting in preferential flow paths for the water phase when these fingers evolve into channels. Outside the channel the trapping of hydrogen is high, while inside the channel an almost perfect sweep occurs. This finger/channel formation could lead to significant trapping of hydrogen during reproduction as part of the hydrogen plume will be bypassed and could potentially have a big impact on the performance of UHS.

To derive meaningful upscaled multiphase flow parameters both the hydrogen and water phases need to flow through the same pore space. As such multiphase flow parameters cannot be accurately derived when gravity segregation and channel flow occurs. Visualization of the saturation distribution is therefore necessary to ensure the validity of relative permeability and capillary pressure measurements for the ${\text {H}}_2$/water system.

## Supplementary Information

Supplementary Information.

## Data Availability

The experimental CT and pressure data are made available open access to the public at https://gitlab.tudelft.nl/ADMIRE_Public/h2_ct_data.
