# Supplementary material for: Experimental characterization of H2/water multiphase flow in heterogeneous sandstone rock at the core scale relevant for underground hydrogen storage (UHS)
Source: Sci Rep. 2022 Aug 26;12:14604. doi: 10.1038/s41598-022-18759-8 (PMC9418204; doi:10.1038/s41598-022-18759-8)
Supplement: Supplementary file 1 — Supplementary Information. [file 41598_2022_18759_MOESM1_ESM.pdf]

Supplementary Information: Experimental characterization of H_2/water multiphase flow in heterogeneous sandstone rock at the core scale relevant for underground hydrogen storage (UHS)

Maartje Boon & Hadi Hajibeygi
m.m.boon@tudelft.nl

Delft University of Technology, Faculty of Civil Engineering and Geosciences, Delft, 2600 GA, The Netherlands

Literature comparision of relative permeabilty and capillary pressure for H_2/water system

Previously, in the literature, the work of Yekta et al. (2018) [1] presented drainage relative permeabilty and capillary pressure curves for the H_2/water system for the Vosges sandstone. The sample had a permeability of 44mD and a porosity of 19%. Furthermore, it consisted predominantly of quartz 81vol% and K-feldspar 17vol%. The Berea sandstone of this study has a permeability of 203mD, a porosity of 19.7% and pre-dominantly consists out of quartz 95vol%. As a result of the differences between the rock samples used in both studies, the relative permeability and capillary pressure curves for the H_2/water system also differ, as can be seen in Figure 1. The relative permeability curve for the Vosges sandstone shows much higher H_2 saturations, and the point where the relative permeability of the H_2 and water phase cross is higher and occurs at higher gas saturations. This indicates less interference of the H_2 and water phase which makes it easier for both phases to flow in the case of the Vosges sandstone. The two low porosity capillary barriers of the Berea sandstone make it more difficult for the H_2 and water phase to flow and could explain the very low relative permeability cross-over point for the Berea sandstone core. The higher gas saturations at the cross-over point in the case of the Vosges sandstone could indicate less water wet conditions, however, the receding contact angles, 21.6° and 34.9° for shallow and deep reservoir conditions, respectively, obtained by fitting the MICP data to the capillary pressure measurements suggest otherwise. The entry pressure for the lower permeable Vosges sandstone is lower than the entry pressure of the Berea, which is unexpected. However, as can be seen from Figure 1, the saturations for the capillary pressure measurements for the Vosges sandstone cover a very small range resulting in a high uncertainty in the fit of the MICP data.

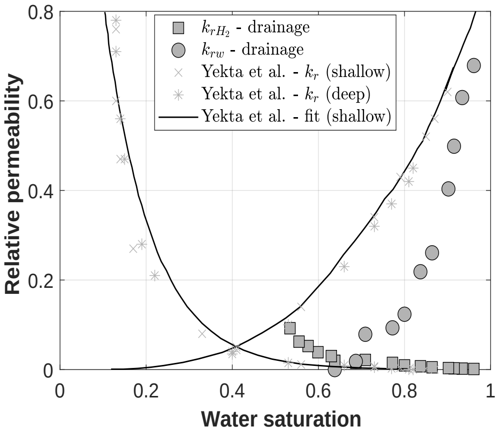

(a)

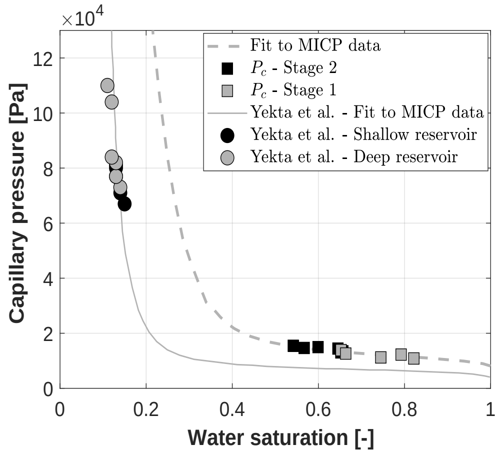

(b)

Figure 1: Comparision of the drainage relative permeability and capillary pressure curves for the H_2/water system presented in this study and the study of Yekta et al. (2018). **(a)** Drainage relative permeability. The circles indicate the water relative permeability measurements while the squares are the H_2 relative permeability measurements of this study. The crosses and stars are the experimental results of the study of Yekta et al. (2018) [1] for the Vosges sandstone for shallow and deep reservoirs, respectively. **(b)** Drainage capillary pressure. The black squares are the capillary pressures measured during stage 2 of this study: Drainage capillary pressure measurements. The grey squares are the capillary pressures measured during Stage 1 of this study: Drainage relative permeability measurements. The dashed line shows the fit to MICP data obtained for a Berea (Liver) sandstone rock core with similar permeability and porosity presented in Ni et al. (2019) [2]. The black and grey circles show the capillary pressures measured in the study of Yekta et al. (2018) for conditions representative of shallow and deep reservoirs, respectively. The grey solid line shows the fit with MICP data for the shallow reservoir results.

References

[1] A. E. Yekta, J.-C. Manceau, S. Gaboreau, M. Pichavant, and P. Audigane, "Determination of hydrogen–water relative permeability and capillary pressure in sandstone: Application to underground hydrogen injection in sedimentary formations," *Transport in Porous Media*, vol. 122, pp. 333–356, 2018.

[2] H. Ni, M. Boon, C. Garing, and S. M. Benson, "Predicting co2 residual trapping ability based on experimental petrophysical properties for different sandstone types," *International Journal of Greenhouse Gas Control*, vol. 86, pp. 158–176, 2019.
